# Physical Frailty and Cognitive Functioning in Korea Rural Community-Dwelling Older Adults

**DOI:** 10.3390/jcm7110405

**Published:** 2018-10-31

**Authors:** Dong Hyun Yoon, Su Seung Hwang, Dong Won Lee, Chung Gun Lee, Wook Song

**Affiliations:** 1 Health and Exercise Science Laboratory, Institute of Sports Science, Seoul National University, 599 Gwanangno, Gwanak-Gu, Seoul 08826, Korea; ycool14@snu.ac.kr (D.H.Y.); duexerss@gmail.com (S.S.H.); liger1987@naver.com (D.W.L.); 2 Department of Psychiatry and Behavioral Science, SMG-SNU Boramae Medical Center, 5-20 Boramaero, Dongjak-Gu, Seoul 07061, Korea; 3 Department of Physical Education, Institute of Sports Science, Seoul National University, Gwanangno, Gwanak-Gu, Seoul 08826, Korea; 4 Institute on Aging, Seoul National University, Seoul 03087, Korea

**Keywords:** cognitive function, disability, physical function, frailty, rural elderly

## Abstract

Cognitive frailty is a heterogeneous clinical manifestation characterized by the simultaneous presence of physical frailty and cognitive impairment. The objective of this study was to investigate the association between physical frailty and cognitive function in rural community-dwelling older Korean adults, taking four cognitive domains into account. We carried out a cross-sectional population-based study which enrolled 104 community-dwelling elderly. Physical frailty phenotype, as well as its individual criteria, were used. Cognitive functioning was examined in the four domains of memory, processing speed, cognitive flexibility, and working memory. Demographic data, lipid profile, muscle strength, physical function, and 25-hydroxyvitamin D (25[OH]D) concentration collected from questionnaire interviews and assessments were included. Of the 104 older adults (77% female), 24.3% were classified as robust, 49.6% as prefrail, and 16.5% as frail. Linear regression analyses showed that the severity of frailty index was associated with four cognitive domains Muscle strength (i.e., Grip strength, Knee extensor and flexor), physical function (i.e., SPPB and Gait speed), and 25[OH]D were associated with poorer cognitive function. Within our population of Korean rural community-dwelling older adults, physical frailty status, muscle strength, physical functions, and biochemical measurements were associated with poorer cognitive function. Synchronicity of physical frailty and cognitive dysfunction may contribute to the negative health-related effects associated with aging.

## 1. Introduction

Frailty is considered as an early stage of disability that is amenable to preventive interventions. Frailty is reversible, while true disability is irreversible. Frailty is one of the most problematical clinical manifestations of aging [1]. A consensus study has defined frailty as a medical syndrome that manifests a critical decline in functional and physiological reserves of multiple organic systems [2]. It is widely accepted that frailty includes shrinking (nutritional/metabolic component assessed by unintentional weight loss), weakness (indicated by muscle strength), poor endurance and energy (per self-reported exhaustion), slowness (demonstrated by slow walking speed), and low physical activity [3]. Whether cognitive domains should be included in frailty is debatable [4]. Concerning pathophysiological mechanisms of frailty, some studies have specifically implicated that low performance in verbal fluency, episodic memory tasks [5], and executive function tests [6] might be mechanisms. Whether frailty is linked to the development of cognitive decline and dementia remains unclear [7]. Recently, the International Academy of Nutrition and Aging (IANA) and the International Association of Gerontology and Geriatrics (IAGG) have summarized cognitive frailty as a heterogeneous clinical manifestation characterized by simultaneous presence of frailty and cognitive impairment (Clinical dementia rating score (CDR) = 0.5) in the absence of dementia characterized by concurrent frailty and potentially reversible cognitive impairment [4]. Additionally, they have emphasized the important role of brain aging in cognitive frailty [2,4].

The link between frailty and cognitive impairment is complex. The biological basis of frailty has not been clearly identified yet. It could reflect cumulative results of pathophysiological modifications caused by concurrent chronic conditions [8], subclinical adaptation of systemic homeostatic mechanisms [9], metabolic modifications, and behavioral factors [5]. Some pathways are shared by frailty and mild cognitive impairment/Alzheimer’s disease (AD). Glucose metabolism, cognitive dysfunction, physical dysfunction, and depression are disorders that might be associated with poor vitamin D status. Previous study has summarized neurobiological evidence, and stated that vitamin D might play a role in brain function.

South Korea is experiencing rapid demographical aging. Frailty in older Korean adults is relevant and significant. In Korea, 10%–16% of older urban-dwelling people are frail while 43−59 percent are pre-frail [10]. However, an institutional model of frailty and geriatric conditions in urban and rural agricultural elderly has not been established yet.

Disparities between urban and rural agricultural elderly populations have been reported in association with health status, community resources, and access to preventive services and health care [11]. Although evidence supports the effectiveness of policy and environmental strategies to prevent frailty and promote health equity, much of this evidence is derived from non-rural settings. Therefore, the objective of this study was to examine the link between frailty and cognitive function in rural community-dwelling older adults considering four cognitive domains. We hypothesize that frailty is linked to poor functioning in the four cognitive domains.

## 2. Materials and Methods

### 2.1. Study Sample

We conducted a cross-sectional population-based study that enrolled 115 community dwelling elderly (≥65 years) Koreans living in rural areas of five of 11 communities in Sunchang Country, Jeonbuk Province, Korea, 282 km south of Seoul. The total population of this region is 29,949; older adults comprise 31 percent of the population.

Between May–December 2015, screening and recruitment was conducted in eight communities. Participants were community-living older adults with the following inclusion criteria: sedentary (less than three hours of weekly physical activity), ability to walk 10 m without a walking aid, and resident of Sunchang Country. Based on previous reports, we excluded participants with a history of AD, stroke, or Mini-Mental State scores < 18; musculoskeletal impairment; presence of psychiatric or neurological disorders; and dyslexia [12]. Cross-sectional data included demographic, frailty index, and neurocognitive characteristics collected from extensive questionnaire interviews and assessment. Eleven were excluded because they had low cognitive function (i.e., MMSE score > 18) or refused lipid measurement. Ethics approval was obtained from the Institutional Review Board of Seoul National University, and each participant provided signed informed consent at enrollment (SNUIRB No. 1512/001-009).

### 2.2. Measurements

#### 2.2.1. Frailty

Frailty was assessed according to the previously criteria [3], and included weight loss, weakness, poor endurance and energy, slowness, and low physical activity level. A person was classified as frail when three or more criteria were met, pre-frail when one or two criteria were met, and robust when none of the criteria was met.

The weight loss of participants was defined according to their answer to the question “Have you ever unintentionally lost as much as 4.5 kg in weight within one year?” [13].

Weakness of participants was defined as low grip strength in each individual corresponding to gender and body mass index (BMI). Grip strength was measured using a hand-to-hand dynamometer (Takei Scientific Instruments, Niigata, Japan). Each participant stood and gripped the hand-to-hand dynamometer handle. Upon verbal command, the handle was gripped as strongly as possible. This was repeated four times with a break in between. The average grip strength in kilograms was recorded [13].

Exhaustion was defined based on the answers to two questions in the Center for Epidemiologic Studies Depression Scale (CES-D): “How often have you ever felt that everything you had done was useless in the last week?” and “How often have you not been in the mood to do everything you had to in the last week” Exhaustion was indicated by responses of “most of the time” and “often” [13].

Slowness was defined as low gait speed in four meters. Three lines were drawn horizontally on the measuring area. The interval between the first and second lines was one meter, and four meters between the second and third lines, for a total of five meters. Each participant stood on the first line and walked to third line immediately when verbal command was given. The time to traverse from the second to the third lines was recorded. A speed <0.8 m/s indicated frailty-related slowness [11].

Low physical activity corresponded to responses to International Physical Activity Questionnaire (IPAQ) items concerning low, middle, and high levels of physical activity. Responses describing low physical activity were indicative of frailty [2]. We used a modified version of the original Cardiovascular Health Study (CHS) frailty index (CHS index), which has been well validated. The subjects having a score of 0 were considered robust, 1–2 indicated prefrail, and ≥3 indicated frail condition [3]

#### 2.2.2. Functional Performance: Short Physical Performance Battery (SPPB)

SPPB was used to assess balance, walking, strength, and endurance. Each test receives a performance score, with a total of 12 points, comprising of the chair stand test (four points), balance test (four points), and a 4 m gait speed test (four points). In the chair stand test the participants were initially seated. On verbal command, they stood up then sat down five times. The time in seconds to complete the task was recorded using a stopwatch. Balance was measured in three tests, following an explanation. In the side-by-side stand test, feet were positioned together and balance was maintained for 10 s. In the semi-tandem stand test, each participant stood with a toe of the dominant foot touching the middle of the opposite foot for 10 s. In the tandem stand test, each participant stood with the toe of the dominant foot touching the heel of opposite foot for 10 s. In the walking speed test, the four-m gait test was used. The average time of two trials of the walking speed test were recorded. A change in SPPB score of 1.0 point was considered significant [12].

#### 2.2.3. Strength Assessment: Leg Muscle Strength

Leg muscle strength was measured using a model 01163 hand-held dynamometer (Lafayette Instrument Company, Lafayette, IN, USA). The target area was the knee extensor and flexor muscles of the dominant lower extremity. Each participant sat on a chair with hip and knees kept flexed at 90° to assess isometric strength of the target area. The chair was leaned against a wall to avoid unintended movement. The technician was positioned on one knee with hands held above each participant’s talotibial joint (anterior part) and lateral malleolus (posterior part) area to assess isometric voluntary contractions of knee extensor and flexor muscles. Each participant was asked to perform two maximal contractions five seconds in duration for each muscle group [14]. After the knee extension, there was a 60-second rest to recover from thigh fatigue. Value of assessment was recorded as the average of two trials. A torque value was derived using an index below [15].

#### 2.2.4. Cognitive Functioning

To assess participants’ cognitive function, a sensitive and validated neuropsychological test battery was used. The cognitive assessment included global cognitive functioning (the Korean version of Mini-Mental State Examination) [16], along with other cognitive measures. This test is commonly utilized to screen for dementia. The test consists of 11 questions and tasks, in a total of five cognitive domains: orientation (10 points), memory (6 points), attention (5 points), language ability (7 points), and comprehensive/judgment (2 points). The highest score is 30 and a higher score indicates a higher level of cognitive function.

Recall and recognition (memory) were assessed using the Rey 15-Item memory test [17]. The test encompasses the memorization of 15 different items (letters, numbers, simple geometric shape) presented in five rows (three items/row). Each participant was shown a paper with 15 different items on it for 10 s. The paper was removed and the participant recorded in writing as many of the items as they could recall. The recognition task scores used two parts.

Processing speed was assessed using the Trail Making A Test [18]. The trail making test consists of two parts. In Part A (TMT-A), the subject was tasked with listing numbers 1–25 in ascending order [18].

Cognitive flexibility was assessed using the Trail Making B Test. In Part B (TMT-B), the subject drew numbers and letters in alternating order. The maximum amount of time to complete Part B was 300 s. TMT-B is more difficult than TMT-A because of an increased demand of motor speed and visual searching [19].

The Digit Span test was used to test working memory. Respondents were asked to recall numbers forward (range 3−9) and backward (range 2−8) [20].

#### 2.2.5. Biochemical Measurement

Blood samples were collected after an overnight fast and stored at −80 °C until the time of analysis. Serum glucose, triglyceride, high-density lipoprotein (HDL), low-density lipoprotein (LDL), and total cholesterol levels were measured using enzymatic colorimetric assays (Roche, Berlin, Germany). Serum 25-hydroxyvitamin D (25[OH]D) levels were measured using a chemiluminescence immunoassay (CLIA, Liaison; Diasorin, Saluggia, Italy).

### 2.3. Statistical Analyses

Statistical analyses were performed using SPSS 22.0 (IBM Corporation, Chicago, IL, USA). Basic characteristics of the study sample were stratified by frailty status. Robust, pre-frail, and frail older patients were compared using the χ^2^ test (categorical variables) and Student *t* test (continuous variables). ANOVA was used to measure the link between each of the four individual domain tests of cognitive performance (processing speed, cognitive flexibility, working memory, and memory) as the dependent variable and each of the several indices of frailty (weight loss, weakness, exhaustion, slowness, and low physical activity) as the independent variable. Regression models were unadjusted results, as well as models adjusted for age, sex, and level of education. Threshold for statistical significance was considered with *p*-value < 0.05.

## 3. Results

At baseline, the mean age of the study participants was 73.5 (±5.43 SD, SD: standard deviation), 77 percent were female, 19 (16.5 percent) were physically frail, and the level of education was 5.1 (±4.17 SD). Table 1 presents the characteristics of robust (24 percent of total), prefrail (49 percent), and frail (16 percent) patients.

The frailty index was significantly correlated to processing speed (*p =* 0.008), cognitive flexibility (*p =* 0.010), working memory (*p =* 0.000), and memory (*p =* 0.004) (Table 2). Of the specific criteria, weight loss was not significantly correlated to cognitive performance, whereas weakness was only significant correlated to working memory (*p =* 0.023). Exhaustion was significantly correlated to processing speed (*p =* 0.001), cognitive flexibility (*p =* 0.009), and working memory (*p =* 0.000). Slowness presented significant correlations with processing speed (*p =* 0.013), working memory (*p =* 0.000), and memory (*p =* 0.001). Low activity revealed only significant correlations with memory (*p =* 0.020) (Table 2).

Grip strength was significantly associated with processing speed (*p =* 0.001), cognitive flexibility (*p =* 0.005), working memory (*p =* 0.000), and memory (*p =* 0.006). Knee extensor and flexor was significantly associated with processing speed (*p =* 0.010, *p =* 0.021; respectively), cognitive flexibility (*p =* 0.008, *p =* 0.007; respectively), working memory (*p =* 0.000, *p =* 0.001; respectively), and memory (*p =* 0.010, *p =* 0.039; respectively) (Table 3).

SPPB was significantly associated with processing speed (*p =* 0.049), working memory (*p =* 0.000), and memory (*p =* 0.004), whereas Gait speed was significantly associated with processing speed (*p =* 0.001), cognitive flexibility (*p =* 0.027), working memory (*p =* 0.000), and memory (*p =* 0.002) (Table 4).

Serum 25-hydroxyvitamin d (25(OH)D) was significantly associated with cognitive flexibility (*p =* 0.038) and memory (*p =* 0.046). In the fully adjusted model, 25[OH]D was not significantly associated with all cognitive performance (Table 5).

## 4. Discussion

A significant association was evident between frailty and cognitive function in 104 rural community-dwelling older adults considering four cognitive domains (processing speed, cognitive flexibility, working memory, memory). To the best of our knowledge, this is the first study to describe health conditions of cognitive function and frailty status of elderly rural residents. Recent reports show that in Western countries, approximately 10% of community-dwelling older adults are frail and 40% are prefrail, while in Korea, 10%–16% are frail and 43%–59% are prefrail in an urban population. However, the burden of frailty status in older Koreans living in rural communities has not been established [11]. Several studies have found that physical phenotype of frailty is associated with the onset of cognitive impairment [2,5,7], consistently revealing a higher prevalence of cognitive impairment among frail older persons. Our cross-sectional analysis also revealed that pre-frail and frail participants overall were more likely to develop cognitive impairment, with nearly 90 percent of frail older adults being cognitively impaired.

A major contribution of this study is the assessment of specific aspects of cognitive function in frail and pre-frail participants. Frailty is linked to worse cognitive function in domains of processing speed, cognitive flexibility, working memory, and memory. These findings expand findings of previous studies on community-dwelling older adults [4]. Recent epidemiological evidence suggests that frailty may increase the risk of future cognitive decline and that cognitive impairment may increase the risk of frailty, suggesting that cognition and frailty may interact with advancing aging [21]. In our study, processing speed, cognitive flexibility, and working memory are considered executive functions.

Recent reports showed strong links between cognition and muscle strength (i.e., grip strength and leg strength), as well as gait speed [2,5,22]. Gait speed and muscle strength are critical components of the frailty phenotype. Grip strength, especially, is a significant marker of sarcopenia. It has been proposed as a single useful marker of generalized frailty and biological aging [23]. In the Hertfordshire Cohort Study, cross-sectional data on community-dwelling men and women age 59–73 have linked lower handgrip strength to poor health-related quality of life [24]. Recent findings from the Aizu Cohort Study suggests a link between baseline handgrip strength and the risk of cognitive disorder in community-based individuals age 40–79 years after a one-year follow-up, showing significant linkage between lower handgrip strength and cognitive disorder [25]. Another study has revealed that decreased muscle strength may be a risk factor for AD. Loss of muscle strength is likely the result of an underlying disease process that leads to cognitive decline and clinical AD [22]. Collectively, these studies consistently suggest that cognitive function is linked to grip and leg strength. Additionally, we have found significant linkage of cognitive function in all domains. On the other hand, we found an association of cognitive function with the continuous variable of grip strength, however, not with the categorical variable of weakness. It is unclear why these contradictory associations were observed; however, this may be explained by the reduction in statistical power for the categorical variable analysis in this small sample (*n* = 104).

In the Irish Longitudinal Study on aging, slow gait as an individual component of frailty phenotype was associated with poor cognition, and prospectively, with incident non-AD dementia in older adults with frailty [26]. Therefore, gait velocity is a strong predictor of adverse events such as disability, mortality, hospitalization, and falling [27]. The cutoff point for walking speed in the present study was 0.8 m/s, which was a critical point for predicting future functional decline in community-dwelling elderly individuals. Recently, findings from the Gait and Brain Study suggested another model of potentially reversible cognitive frailty incident rate, though not risk for progression to dementia, although the combination of slow gait and cognitive impairment posed the highest risk for progression to dementia [28]. These results suggest that walking speed may be the most useful measurement for determining frailty and predicting future functional decline in older adults [28]. In this study, links with frailty were driven by gait speed and grip strength. Knee extensor/flexor and SPPB also contributed significantly to links found. Slow low gait, grip strength, and cognitive impairment individually presented risk, while a combination of them increased the risk.

Although several studies have reported that frailty and impaired cognitive functioning are linked, there are currently no causal links [4]. Potential mechanisms that may underlie frailty and cognitive impairment include neuropathological changes, hormonal changes, vascular damage, chronic inflammation, nutritional factors, vitamin D deficiency, and increased insulin resistance [5]. The extent to which similar mechanisms affect frailty and cognition may be different. Previous studies have reported that these links with cognition and frailty may result from a role of vitamin D in the brain and physical function. Many brain cell types and regions can synthesize an active form of vitamin D, including the hypothalamus and substantia nigra [29], which are areas implicated in the pathophysiology. Vitamin D modulates the production of neuroprotective factors and neurotransmitters, neuronal apoptosis, neuro inflammation, oxidative stress, excitotoxicity, and myelin and axon repair [29]. Poor physical performance and muscle weakness have been linked to low 25(OH)D concentration among older adults in a cross-sectional study [30]. In our study, it was found that 25(OH)D is associated with cognitive flexibility and memory.

## 5. Conclusions

In conclusion, in our population of rural community-dwelling older Korean adults, frailty status, muscle strength, physical function, and biochemical measurements were linked with poor cognitive function. Synchronicity of frailty and cognitive dysfunction may be the basis of negative health-related effects associated with aging. Future studies are needed to assess brain β-amyloid deposition of common brain network that regulates frailty and cognitive processes to clarify potential mechanisms underlying these cognitive frailty associations. However, causal links between physical frailty and cognitive impairment are currently unclear. Therefore, longitudinal studies are required to determine associations between cognitive and functional decline in older adults.

## Figures and Tables

**Table 1:** Baseline characteristics for full sample and stratified by frailty status.

Variable	Full Sample, *n* = 104	Baseline Physical Frailty Status			*p* Value
		Robust, *n* = 28 (24.3%)	Prefrail, *n* = 57 (49.6%)	Frail, *n* = 19 (16.5%)	
**Demographics:**					
Age, mean (SD)	73.5 (5.43)	72.4 (5.52)	73.3 (5.53)	75.2 (4.94)	0.270
Female, *n* (%)	80 (77)	20 (72)	42 (74)	18 (95)	0.120
Education, year, mean (SD)	5.1(4.17)	6.2 (3.36)	5.5 (4.48)	2.0 (3.05)	0.012
**Lipid profile:**					
Glucose (mg/dL)	102.8 (24.98)	97.0 (17.66)	105.7 (30.00)	102.8 (15.05)	0.320
Total cholesterol (mg/dL)	179.2 (38.23)	187.5 (46.22)	174.3 (33.50)	181.4 (38.18)	0.316
Triglyceride (mg/dL)	124.2 (29.90)	132.4 (50.85)	117.8 (52.09)	131.1 (40.12)	0.370
LDL-C (mg/dL)	105.86 (35.03)	112.1 (43.23)	102.9 (29.06)	105.6 (38.68)	0.528
HDL-C (mg/dL)	52.74 (14.28)	54.7 (15.76)	51.4 (13.25)	54.0 (15.30)	0.558
**Frailty criteria, *n* (%):**					
Slow gait velocity	17 (16.3%)	0 (0%)	1 (1.75%)	16 (84.21%)	0.000
Shrinking	18 (17.3%)	0 (0%)	11 (19.30%)	7 (36.84%)	0.010
Weakness	26 (25%)	0 (0%)	13 (22.81%)	13 (68.42%)	0.000
Exhaustion	50 (48.1%)	0 (0%)	33 (57.89%)	17 (89.47)	0.000
Low activity level	31 (29.8%)	0 (0%)	18 (31.58%)	13 (68.42%)	0.000
**Cognitive functioning:**					
Global cognitive functioning (MMSE), mean (SD)	23.5 (2.43)	24.4 (2.75)	23.3 (2.10)	21.9 (2.02)	0.015
Cognitive impairment (MMSE < 23), *n* (%)	66 (64%)	12 (42.86%)	37 (64.91%)	17 (89.47%)	0.006
Memory, mean (SD)	19.3 (6.23)	21.3 (4.41)	19.7 (6.30)	12.9 (6.31)	0.004
Processing speed, mean (SD)	75.4 (41.91)	57.5 (25.99)	79.1 (42.54)	109.0 (54.92)	0.012
Cognitive Flexibility, mean (SD)	204.8 (78.30)	171.6 (69.28)	215.1 (79.42)	259.3 (61.47)	0.026
Working memory, mean (SD)	8.20 (2.67)	9.8 (1.72)	8.1 (2.57)	6.1 (2.66)	0.000

HDL: High density lipoprotein; LDL: Low density lipoprotein; MMSE: Mini-Mental State Examination. SD: standard deviation.

**Table 2:** Association between presence of each criterion of the frailty index and different measures of cognitive function (fully adjusted).

	Processing Speed		Cognitive Flexibility		Working Memory		Memory	
	*β*	*p*	*β*	*p*	*β*	*p*	*β*	*p*
**Frailty index** *								
Unadjusted	0.35	0.008	0.34	0.010	−0.41	0.000	−0.34	0.004
Fully adjusted	0.34	0.035	0.39	0.020	−0.38	0.003	0.19	0.029
	**Mean(SD)**	***p***	**Mean(SD)**	***p***	**Mean(SD)**	***p***	**Mean(SD)**	***p***
**Weight loss**								
Yes	57.00(24.8)	0.186	174.00(61.3)	0.271	7.66(2.7)	0.142	19.50(7.0)	0.878
No	78.17(43.4)		208.98(79.8)		8.57(2.2)		19.12(6.2)	
**Weakness**								
Yes	92.63(50.6)	0.131	232.58(74.3)	0.171	7.45(2.5)	0.023	16.53(6.2)	0.089
No	71.46(39.2)		197.74(78.4)		8.71(2.2)		19.87(6.1)	
**Exhaustion**								
Yes	94.85(48.8)	0.001	234.23(77.8)	0.009	6.50(1.6)	0.000	17.85(7.2)	0.146
No	59.39(26.7)		181.66(71.5)		8.77(2.3)		20.20(5.3)	
**Slowness**								
Yes	112.00(52.2)	0.013	249.50(58.3)	0.142	7.07(1.1)	0.000	12.50(5.6)	0.001
No	70.50(38.3)		199.77(79.0)		9.04(2.2)		20.16(5.7)	
**Low activity**								
Yes	89.81(44.2)	0.108	220.21(81.4)	0.404	8.06(2.1)	0.274	16.17(6.5)	0.020
No	70.09(40.2)		200.04(77.5)		8.63(2.4)		20.31(5.8)	

β, completely standardized regression coefficient. *, CHS Frailty index: 0 = robust, 1−2 = pre-frail, 3−5 = frail [3]. Adjusted for age, sex, level of education. SD, standard deviation.

**Table 3:** Association between grip strength and leg strength with measures of cognitive function. (fully adjusted).

	Processing Speed		Cognitive Flexibility		Working Memory		Memory	
	*β*	*p*	*β*	*p*	*β*	*p*	*β*	*p*
**Grip strength (kg)**								
Unadjusted	−0.45	0.001	−0.37	0.005	0.42	0.000	0.36	0.006
Fully adjusted	−0.32	0.017	−0.30	0.833	0.24	0.026	0.25	0.042
**Knee extensor (Nm)**								
Unadjusted	−0.34	0.010	−0.36	0.008	0.43	0.000	0.34	0.010
Fully adjusted	−0.23	0.119	−0.09	0.543	0.21	0.058	0.27	0.046
**Knee flexor (Nm)**								
Unadjusted	−0.31	0.021	−0.36	0.007	0.36	0.001	0.27	0.039
Fully adjusted	−0.11	0.406	0.01	0.915	0.09	0.375	0.17	0.143

β, completely standardized regression coefficient. Adjusted for age, sex, level of education.

**Table 4:** Association between physical function and gait speed with measures of cognitive function. (fully adjusted).

	Processing Speed		Cognitive Flexibility		Working Memory		Memory	
	*β*	*p*	*β*	*p*	*β*	*p*	*β*	*p*
**SPPB (score)**								
Unadjusted	−0.27	0.049	−0.22	0.105	0.46	0.000	0.37	0.004
Fully adjusted	−0.11	0.480	−0.08	0.632	0.30	0.015	0.21	0.136
**Gait speed (s)**								
Unadjusted	0.44	0.001	0.30	0.027	−0.42	0.000	−0.40	0.002
Fully adjusted	0.30	0.063	0.18	0.253	−0.22	0.068	−0.23	0.119

β, completely standardized regression coefficient. Adjusted for age, sex, level of education.

**Table 5:** Association of serum 25-hydroxyvitamin d with measures of cognitive function. (fully adjusted).

	Processing Speed		Cognitive Flexibility		Working Memory		Memory	
	*β*	*p*	*β*	*p*	*β*	*p*	*β*	*p*
**25(OH)D (ng/mL)**								
Unadjusted	−0.25	0.057	−0.28	0.038	0.06	0.535	0.26	0.046
Fully adjusted	−0.01	0.964	−0.02	0.908	−0.18	0.068	0.00	0.989

β, completely standardized regression coefficient. 25(OH)D, 25-hydroxyvitamin D. Adjusted for age, sex, level of education.
